# Structured diabetes care leads to differences in organization of care in general practices: the healthcare professional and patient perspective

**DOI:** 10.1186/1472-6963-11-113

**Published:** 2011-05-23

**Authors:** Andrea S Fokkens, P Auke Wiegersma, Klaas van der Meer, Sijmen A Reijneveld

**Affiliations:** 1 Department of Health Sciences, University Medical Center Groningen, University of Groningen, the Netherlands; 2 Department of General Practice, University Medical Center Groningen, University of Groningen, the Netherlands

**Keywords:** diabetes mellitus, quality of healthcare, patient care team, patient education

## Abstract

**Background:**

Care for patients with chronic diseases is challenging and requires multifaceted interventions to appropriately coordinate the entire treatment process. The effect of such interventions on clinical outcomes has been assessed, but evidence of the effect on organization of care is scarce.

The aim is to assess the effect of structured diabetes care on organization of care from the perspective of patients and healthcare professionals in routine practice, and to ascertain whether this effect persists

**Methods:**

In a quasi-experimental study the effect of structured care (SC) was compared with care-as-usual (CAU). Questionnaires were sent to healthcare professionals (SC n = 31; CAU n = 11) and to patients (SC n = 301; CAU n = 102). A follow-up questionnaire was sent after formal support of the intervention ended (2007).

**Results:**

SC does have an effect on the organization of care. More cooperation between healthcare professionals, less referrals to secondary care and more education were reported in the SC group as compared to the CAU group. These changes were found both at the healthcare professional and at the patient level. Organizational changes remained after formal support for the intervention support had ended.

**Conclusion:**

According to patients and healthcare professionals, structured care does have a positive effect on the organization of care. The use of these two sources of information is important, not only to assess the value of changes in care for the patient and the healthcare provider but also to ascertain the validity of the results found.

## Background

Care for patients with chronic diseases such as asthma, hypertension, and diabetes is challenging and requires multifaceted interventions in order to appropriately coordinate the entire treatment process [1]. Attempts have been made to organize care in such a way that it can meet the high demands of chronic care, using various care models and interventions [1-3]. In many studies only the effect at patient level, such as clinical outcomes, was determined [2,4-7]. Improvements were found in clinical outcomes [5,7,8], and in the proportion of patients having the required laboratory and/or physical examinations [6,9-14]. A multifaceted approach mostly seems to lead to improvements at patient level [1,3,15].

An assessment of the effectiveness of an intervention should assess the effect not only at the patient level but also at the level of the healthcare professional [16-18], because their perspectives about organization of care can differ widely. This is relevant in order to appropriately assess instant outcomes but also to determine the likelihood of a longer lasting effect. For the latter, the perspective of the healthcare professional is particularly relevant.

Moreover, the use of a care-as-usual group (CAU) is needed to appropriately assess the effectiveness of organizational interventions. In rapidly changing health-care delivery systems, the assumption that "usual care" will be static is most likely to be mistaken [19]. The changes found may, for instance, be due to a general improvement in quality of care that occurred independently of the intervention under study. The impact on the organization of care should be determined, and the inclusion of a control group is needed to figure out whether such general improvements have indeed occurred. Few diabetes intervention studies take the effect on organization of care into account, both in an intervention as well as in a control group.

The aim of this study is to assess the effect of structured diabetes care on the organization of care according to the perspective of patients and healthcare professionals in day-to-day practice, and to assess whether this effect persists. To determine the degree to which the organization and components of care have changed due to the intervention, we compared reports of patients and healthcare professionals between the SC and CAU control groups. Data were also collected one year after formal support for the intervention had ended.

## Methods

### Design

This study was a quasi-experimental study to determine whether the implementation of SC led to differences in organization of care compared with CAU. For this, questionnaires for both healthcare professionals and patients were used. Data were also collected one year after the formal support for the intervention had ended.

### Study population

General practices in the north of the Netherlands were asked to participate in the SC programme from the beginning of 2003. At the time of data retrieval the SC group consisted of 45 practices. For the CAU group, practices were eligible if they did not participate in a diabetes-specific care improvement programme and if they were located in a region comparable to that of the structured care group. This CAU group consisted of 14 general practices that took part in another unrelated effect study. The intervention in that effect study can not have affected our study because it was just started after the completion of our data collection. In the SC group, data were available from twenty diabetes type 2 patients per practice and in the CAU group from fifteen diabetes type 2 patients per practice. In both groups the patients were randomly selected from the electronic medical record in the general practice.

### Intervention

**Figure 1:**
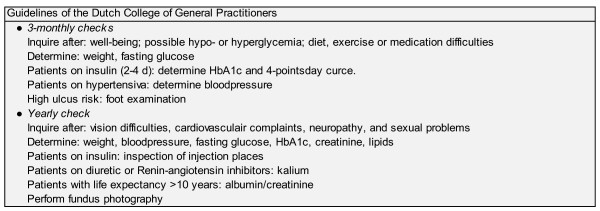
Guidelines of the Dutch College of General Practitioners

**Figure 2:**
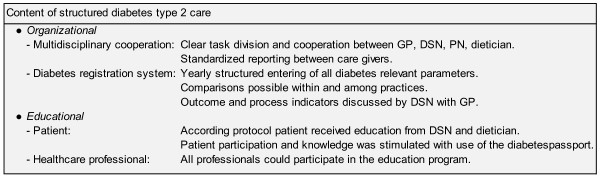
Content of structured diabetes type 2 care

The Structured Care was an initiative of the local health insurance company, hospital, domiciliary care and general practitioners. The Structured Care contents were established in different phases (choice of intervention; identification of components, protocol and outcomes; effect study) [20] in close cooperation with the different healthcare providers. The goal of the SC was to establish comprehensive and efficient care for type 2 diabetes patients in a primary care setting [21]. The care was organized in accordance with the national clinical guidelines of the Dutch College of General Practitioners (Figure 1) [22,23] and enhanced with a number of organizational and educational components. Organizational aspects consisted of multidisciplinary cooperation, a clear task division and cooperation between the general practitioner (GP), the diabetes specialized nurse (DSN), the practice nurse and the dietician (Figure 2). The general practitioner remained responsible for the diabetes care in the entire practice population. As part of the intervention the following patients were referred to a dietician or a diabetes specialized nurse; all patients on insulin with a dosage adjusted longer than 12 months ago who had not visited a DSN in the meantime; all patients who had not visited a DSN or dietician for three years and over; patients with poorly controlled DM; all patients for whom either the GP or the PN or the patient judged a contact to be necessary.

Every patient has at least one diabetes check every 12 months, among others (Figure 1) blood glucose, blood pressure, and feet is examined (examination of feet for ulcers, sensory perception and arterial pulse). Standardized reporting was used between the different health care professionals.

In addition, all relevant clinical parameters were registered in a structured registration program called Diabcare [24], and used for comparisons within and between practices [25]. The DSN discussed these parameters with the general practitioner on an annual basis.

The educational component targeted both patients and healthcare professionals. The patients received individual education from a DSN and a dietician. In addition, they received a "Diabetes Passport" to record medication, laboratory results, treatment targets and personal information. The healthcare professionals took part in an education programme consisting of lectures on a number of relevant topics such as the diabetic foot, neuropathy and diet. The SC programme was formally supported for four years.

### Care-as-usual (CAU)

The practices in the control group provided diabetes care according to care-as-usual. This care was based on the national guidelines of the Dutch College of General Practitioners, and consisted of four checks per year, involving three general checks and one more extensive check a year (Figure 1) [22,23]. Checks were performed by the GP, practice nurse, and/or practice assistant.

### Data collection

A questionnaire was sent to the healthcare professionals and patients in the SC and CAU group. The healthcare professionals (GP, practice nurse and DSN) were asked questions about the organization of care. For each of the practices that the DSN cooperated with, the nurse was able fill in separate answers.

The questionnaire for the patients consisted of questions about the care they received; contacts, examinations and education. The cooperating practices (SC and CAU) sent the questionnaires to the patients in 2006. This was two or three years after the start of the SC, depending on the year the practice entered the SC programme. From 2007 on the SC association support ended. After one year the questionnaire was sent again to those patients who had indicated their willingness to take part in the follow-up (by returning their mailing addresses). Additional information was retrieved from the SC practices regarding the organization of care when the SC support ended.

The Medical Ethics Committee agreed on study design.

### Statistical methods

First, response rates and characteristics of practices and patients were determined for the SC and CAU groups. Next, comparisons were made between the different aspects of organization of care of the SC and CAU groups. A two-sample t-test or Mann-Whitney test was used for continuous variables and Pearson Chi-Square or Fisher's exact test was used for categorical variables. The results of the follow-up questionnaire were compared with the first questionnaire between and within the SC and the CAU groups using the McNemar test, the Pearson Chi-Square or Fisher's exact test. Statistical analyses were performed with SPSS 14.0. P-values < 0.05 were considered to be significant.

### Ethical approval

The Medical Ethics Committee agreed on study design

## Results

### Description of the sample

The questionnaires for the healthcare professional were returned by 31 (69%) practices in the SC group and by 11 (79%) in the CAU group. The practices in the SC and CAU groups did not differ regarding background characteristics (Table 1 upper part). The questionnaires for patients were returned by 301 (43.2%) patients in the SC group and 102 (50.7%) in the CAU group. Patients in the CAU group had a longer duration of diabetes, a higher proportion of patients used insulin, and a higher proportion of patients had cardiovascular complications (Table 1 lower part). The higher proportions of patients that used insulin in the control group could not be explained by the longer duration of diabetes or age.

**Table 1:** Characteristics of practices and patients

Practices	Structured Care	Care-As-Usual
N	31	11
Single, n (%)	15 (48.4)	5 (45.5)
Duo, n (%)	8 (25.8)	5 (45.5)
Group, n (%)	8 (25.8)	1 (9.1)
Practice nurse employed in practice (%)	64.5	63.6
Patients per practitioner, mean (sd)	2161 (730)	1872.5 (603)
Diabetes patients per practitioner, mean (sd)	78 (37.3)	53.9 (30.3)
Patients treated by internist, mean % (sd)	11.0 (8.4)	11.2 (4.9)

Patients		

N	301	102
Age, years, mean (sd)	65.0 (11.2)	66.9 (9.0)
Male, % (N)	51.5 (155)	50.0 (51)
Duration of diabetes (years)	6.2* (6.3)	9.9* (8.2)
Insulin therapy, % (N)	10.5* (31)	38.2* (39)
Diabetes related complications		
Cardiovascular % (N)	15.8* (42)	29.9* (26)
Eye % (N)	19.4 (54)	19.3 (17)
Foot % (N)	13.5 (37)	11.5 (10)
Renal % (N)	4.5 (12)	3.5 (3)
Neuropathic % (N)	5.2 (14)	4.5 (4)
Country of origin the Netherlands % (N)	95.7 (288)	95.1 (97)

* p < 0.05, between Structured Care and care-as-usual group.

Percentages may not add up to 100% due to missing data

Follow-up rates for the patients after one year were 189 (62.8%) and 61 (59.8%) respectively. The follow-up questionnaire responders and non-responders were comparable in age, gender and had the same duration of diabetes.

### Information from healthcare professionals

No differences were found between the SC and CAU groups in yearly and tri-monthly checks; these were mainly performed by the practice nurse (Table 2). A statistically significant difference was found for healthcare professionals who provided insulin therapy, being mainly the DSN and the GP in the SC group and the internist in the CAU group (Table 2).

**Table 2:** Components of care provided according to the healthcare professional

	Structured Care (31)	Care-As-Usual (11)	*P *-value
Yearly check performed by (%)			
General practitioner	29.0	36.4	0.65
Practice nurse	51.6	63.6	0.49
Practice assistant	6.5	0	0.39
Diabetes specialist nurse	3.2	0	0.55
GP with PN/PA	9.7	0	0.28
Tri-monthly checks performed by (%)			
General practitioner	16.1	9.1	0.57
Practice nurse	61.6	63.6	0.89
Practice assistant	22.6	27.3	0.75
Insulin treatments provided by (%)			
Internist	6.5**	63.6**	<0.001
General practitioner	61.3	36.4	0.15
Practice nurse	16.1	18.2	0.88
Diabetes specialist nurse	67.7**	18.2**	0.005
Consultation between healthcare professionals (%)			
General practitioner	100*	80.0*	0.02
Practice nurse	71.4	70.0	0.93
Practice assistant	46.4	40.0	0.73
Diabetes specialist nurse	78.6**	30.0**	0.005
Dietician	50.0*	10.0*	0.03
Estimated percentage of patients that received education			
Diet % (sd)	86.7 (17.9)	83.5 (28.7)	0.91
Exercise % (sd)	89.6 (16.0)	76.0 (29.5)	0.17
Smoking behavior % (sd)	90.0 (11.4)	73.3 (44.2)	0.17
Foot examination % (sd)	89.3 (17.0)	69.5 (38.3)	0.14
Education on diet, exercise, foot examination or smoking behavior.			
by DSN (%)	35.0*	0.0*	0.04
by GP (%)	61.3	81.8	0.24
by PN/PA (%)	87.1	90.0	0.74
Diabetes passport, often or always used in practice (%)	78.6*	40.0*	0.05


* p < 0.05, ** p <0.01, between Structured Care (SC) and care-as-usual (CAU) group.

#### Multidisciplinary cooperation

In the SC group, the DSN, the dietician, and the general practitioner were involved in a consultation with other healthcare professionals in the SC group significantly more often than in the CAU group (Table 2).

#### Registration and education

The healthcare professionals in the SC group used the diabetes passport significantly more often than those in the CAU group (Table 2). The estimated percentage of patients who received education was higher in the SC group than the CAU group, but this did not reach statistical significance. Patients' reports on education received were significantly higher in the SC group (Table 3). This education was significantly more often provided in the SC group by the DSN and less often by the GP (Table 2). Finally, all practices in the SC group used the diabetes registration system. When the formal support for SC ended, the practices mostly kept to their changed organization of care, but only 11 (32%) practices continued using the registration program Diabcare.

**Table 3:** Components of care received according to the patient

	First questionnaire			Second questionnaire		
	**SC^# ^** **(301) **	**CAU^## ^** **(102) **	***P *-value **	**SC^# ^(189) **	**CAU^## ^(61) **	***P *-value **

Contact healthcare professional during the past year, % (N)						
General practitioner	59.0*(177)	46.1*(47)	0.02	58.5*(110)	43.5*(27)	0.04
Diabetes specialist nurse	37.5 (112)	43.1 (44)	0.31	38.5 (73)	51.6 (32)	0.08
Practice nurse	34.0**(102)	17.6**(18)	0.002	35.1*(66)	16.1*(10)	0.05
Practice assistant	24.1* (72)	13.7* (14)	0.03	26.6*(50)	12.9*(8)	0.03
Dietician	26.3* (79)	15.7* (16)	0.03	18.1(34)	11.3(7)	0.21
Ophthalmologist	72.6 (217)	68.6 (70)	0.45	75.0(141)	80.6(50)	0.40
Internist	9.7** (29)	44.1**(45)	<0.001	11.2**(21)	41.9**(26)	<0.001
Cardiologist	13.0 (39)	18.0 (18)	0.22	11.7(22)	16.1(10)	0.38
Received good education on % (N):						
Nutrition/diet	94.3* (265)	85.9* (79)	0.01	94.3*(165)	82.2*(48)	0.01
Exercise	91.0 (243)	85.6 (77)	0.16	94.5**(156)	80.4**(45)	0.001
Smoking	71.7 (114)	60.3 (38)	0.07	77.3(75)	65.8(25)	0.19
Foot care	89.5**(247)	69.9**(65)	<0.001	93.6**(41)	74.5**(160)	<0.001
Checks/examination during the past year of % (N):						
Blood pressure	99.4 (295)	97.5 (96)	0.07	100(186)	100(61)	-
Weight	97.5**(276)	89.9**(88)	<0.001	98.9*(182)	91.7*(55)	0.01
Eye	89.5 (246)	89.0 (86)	0.88	91.8(169)	93.0(53)	0.99
Foot	92.4**(253)	76.2**(68)	<0.001	96.0**(168)	82.8**(53)	0.002
Diabetes passport received	66.9**(194)	19.1**(17)	<0.001	70.0**(126)	38.6**(22)	<0.001
HbA1c knowledge	70.9*(205)	56.2* (54)	0.01	74.7*(133)	61.7*(37)	0.05
						

* p < 0.05,** p <0.01, between Structured Care and care-as-usual group. *Percentages may not add up to 100% due to missing data *

^# ^SC = Structured Care ^## ^CAU = Care-As-Usual

#### Satisfaction

The GPs in the SC group were significantly more often satisfied with the organization of diabetes care than in the CAU group (97.4% vs. 72.7%, *p = 0.03) *. No differences on this aspect were found for the practice nurses and practice assistants. In the SC group, GPs were more often of the opinion that there was a structured cooperation among the different disciplines of healthcare professionals (87.2% vs. 63.6%, *p = 0.09) *.

### Information from diabetes specialist nurses

We received information from three DSNs working in the structured care group, who provided information on 27 (60%) practices. In 16 of these practices a practice nurse was employed and in 11 there wasn't one. The DSNs estimated that in 12 practices (44.4%) half or more of the patients were referred to the DSN, in 9 of these practices (56.2%) a practice nurse was employed and in 3 practices (27.3%) not. This is a discrepancy because according to the DSNs, referral was less frequent in those practices with a practice nurse. Reasons given for referral consisted of poorly regulated patients (70.4%), complex patients (51.9%), insulin therapy (55.0%), yearly check (37.5%), and newly diagnosed patients (14.8%).

### Information from patients

Table 3 shows that in the SC group a significantly higher percentage of patients reported contact with the GP, the practice nurse, the practice assistant and the dietician during the past year. A significantly lower percentage of patients reported contact with the internist in the SC group than in the CAU group. A significantly higher proportion of patients in the SC than in the CAU group reported that they received good education about nutrition/diet, and foot care, and they reported knowing their blood glucose level. Also check of body weight and a foot examination was reported by a significantly higher proportion of patients in the SC group.

When insulin users and non-users were analyzed separately, almost all the differences between the SC and CAU remained. Only, the difference in reported contact with the general practitioner was smaller and not significant: 59% of the non-insulin users reported contact in the SC group compared to 52% in the CAU group. For insulin users this percentage was 58.1% in the SC group and 36% in the CAU group.

One year after the SC support ended, the effect of the structured care was still visible. In the SC group, there were no differences between the first and second questionnaire. In the CAU group, there was an increase in foot examination (82.8%, *p = 0.04 *) and in percentage patients that reported to have received a diabetes passport (38.6%, *p = 0.01) *. However, the differences were still significant in favour of the SC group (Table 3).

## Discussion

Our results show that, according to patients and healthcare professionals, structured care has a positive effect on the organization of care in routine practice. In the SC group insulin therapy was provided mostly in primary care, in the CAU group it was provided in secondary care by the internist. Furthermore, consultation between healthcare professionals occurred more often in the SC group. We also found that education was reported more frequently in the SC group as compared to the CAU group. The education in the SC group was more often provided by the DSN and resulted in better knowledge of blood glucose levels by the patients. After a year the effect was still visible, meaning that even without formal SC support the practices were able to maintain the structured care on their own.

Our findings show that an intervention in the organization of chronic care has a positive effect according to the healthcare professionals whereas until now primarily clinical outcomes have been the object of study [13,2]. Some previous studies did determine the impact on the organization of care. They showed that team effectiveness [26] and use of chronic care model elements [27] contributed to improving quality of care. However, Bosch et al. did not find associations between organizational factors and clinical outcomes [28]. Contrary to our study, however, these studies did not take patient perspectives into account. Nutting et al. [27] did, and they found an association between the use of chronic-care model elements and patient opinion about support from the practice.

The goal of SC was to provide comprehensive and efficient diabetes primary care; this was established by enhancing routine care with organizational and educational components. We found an effect on the organizational component (more cooperation) and educational component (more education). However, the structured monitoring of outcomes by using a registration programme did not come up to expectations. This registration programme was used by all the practices, but only 32% continued using the program after SC support stopped. The balance between data entry and extraction possibilities is important; for a registration program to be used in practice it must meet the demands of the users [29]. The registration program may be too intricate for long term use in routine practice. Nevertheless compliance was relatively high; in another study that determined the use of intervention by interviewing clinicians, only 19% reported that they were in fact regularly putting the approach into practice [30]. A user-friendly registration programme for efficient patient care and care management deserves additional attention in interventions. Research and development of a registration program that can meet the demands of the health care professionals and has easy data management possibilities is necessary.

### Strengths and limitations

One strength of this study is that the different aspects of organization of care were taken into account using the patients' and the healthcare professionals' perspectives. Other strengths were its embedding in routine practice, the comparability of the practices in the SC and CAU groups and the high response rates among healthcare professionals. The percentages of questionnaires that were returned by the patients were relatively low, i.e. 43.2% and 50.7% for SC and CAU, respectively. This may have led to response bias, with for instance less motivated patients with more problems responding less. However, this is likely to have occurred in both the SC and CAU group. This limits the likelihood that response bias has affected the results of the comparisons that we made.

Another limitation of our study was the difference between the SC and CAU groups regarding insulin use of patients. The most likely explanation may simply be chance as the insulin use reported by the practices themselves was comparable, and also in line with insulin use cited in the literature [31,32]. However, almost all differences between the SC and CAU groups remained when reanalysing the data separately for insulin and non-insulin users. Therefore, the difference in insulin use between the two groups does not seem to have had an impact on the results of our study. Another possible limitation was the small number of DSNs in the SC group. This reflects, however, routine care in which one DSN serves a number of practices. Particularly regard to the DSN perspective, our results thus need confirmation in future studies.

### Implications

The aim of our study was to determine the effect of structured diabetes care on organization of care according to the perspective of patients and healthcare professionals in day-to-day practice, and to ascertain whether this effect persists. Our findings imply that SC has positive effect on the organization of care and, there-fore implementation can be considered in routine practice. Significantly more cooperation, more care within primary care and more education were found in the SC group as compared to the CAU. These changes were found at the level of both healthcare professionals and patients.

Changes in the organization of care also remained after support for the SC intervention stopped. In the long-term, this implies that structured care can be maintained. Again this supports the assumption that SC should be seriously considered for the improvement of care for patients with chronic diseases in order, to coordinate the entire treatment process. However, further research, with bigger samples and in other chronic diseases is needed to confirm our findings.

A cost-effectiveness analysis is necessary to determine the impact of Structured Care on costs. Cost reduction is possible when the workload of general practitioner is reduced by multidisciplinary cooperation, also patient complications can be reduced. However, increased use of resources and longer consultation times might offset the savings.

Taking into account the perspective of both the patient and the healthcare professional in this study enabled a complete overview of the effect of structured care on the organization of the care as provided. Both types of informants reported important and lasting positive effects. Especially in care systems, the use of these two sources of information is important, not only to assess the value of changes in care for the patient and the healthcare provider but also to ascertain the validity of the results found.

## Conclusions

According to patients and healthcare professionals, structured care has a positive effect on the organization of care. Changes in the organization of care remained after support for the SC intervention stopped. Significantly more cooperation, more care within primary care and more education were found in the SC group as compared to the CAU.

## Competing interests

The authors declare that they have no competing interests.

## Authors' contributions

All four authors made a substantial and significant contribution during the preparatory phase of this manuscript. They have all approved the final manuscript, and accept full responsibility for the design and conduct of the study; they had access to the data and approved the submission of this paper.

## Pre-publication history

The pre-publication history for this paper can be accessed here:

http://www.biomedcentral.com/1472-6963/11/113/prepub
